# Electric-Field-Assisted Enrichment and Electrochemical Detection of Gram-Positive Bacteria Using an FEM-Guided Interdigitated Electrode Biosensor

**DOI:** 10.3390/bios16090527

**Published:** 2026-09-21

**Authors:** Zeeshan, Naeem Iqbal, Jaeyoung Choi

**Affiliations:** 1 School of Computing, Gachon University, Seongnam-si 13120, Gyeonggi-do, Republic of Korea; 2 Centre for Secure Information Technologies, Queen’s University Belfast, Belfast BT3 9DT, UK; n.iqbal@qub.ac.uk; 3 Momentum 1.0 Research, Queen’s University Belfast, Belfast BT3 9DT, UK

**Keywords:** positive dielectrophoresis (pDEP), electrochemical biosensor, gram-positive bacteria, wave-shaped interdigitated electrode (W_IDE), finite-element modeling (FEM), pDEP-assisted bacterial detection, passive bacterial detection

## Abstract

Dielectrophoretic (DEP) enrichment is a powerful strategy to enhance bacterial capture efficiency and accelerate the response of electrochemical biosensors by actively concentrating target bioparticles at the sensing interface. In this study, a combined numerical–experimental framework is developed to rationally design a DEP-assisted electrochemical biosensor with improved bacterial capture and detection performance. A finite-element modeling (FEM) approach was used to model the coupled electric field, dielectrophoretic force, and particle-transport phenomena, providing a quantitative basis for comparing bacterial trapping efficiency across different interdigitated electrode geometries. The modeling reveals that a wave-shaped interdigitated electrode (W_IDE) generates extended high-field regions and an enlarged effective capture area, resulting in an improved bacterial capture efficiency of 19% compared to 12% for the conventional rectangular interdigitated electrodes (R_IDE) under positive DEP (pDEP) conditions. Based on these insights, the W_IDE was fabricated on a printed circuit board (PCB) substrate, modified with platinum-black (Pt-black) to increase electroactive surface area, and interfaced with a custom-built 16-channel portable potentiostat unit, enabling sequential impedance measurements. The developed biosensor was applied to pDEP-assisted impedance detection of *Staphylococcus aureus* and *Micrococcus luteus* using vancomycin as the capture probe. The pDEP-assisted operation enabled rapid and highly sensitive detection down to 10 CFU/mL within 30 min with a wide linear range (10–10^5^ CFU/mL) in 0.1× PBS, outperforming passive detection (10^2^–10^5^ CFU/mL) for both *Staphylococcus aureus* and *Micrococcus luteus*. In skim milk, however, the linear detection ranges shifted to 10^2^–10^5^ CFU/mL with pDEP-assisted detection and 10^3^–10^5^ CFU/mL under passive detection conditions. Overall, this work highlights the significance of combining FEM-optimized electrodes with DEP-driven enrichment to achieve improved bacterial capture, sensitivity, and robustness in electrochemical biosensors.

## 1. Introduction

The World Health Organization (WHO) identifies bacterial infections as the second leading cause of death globally, following heart disease. In 2019, these infections accounted for approximately 7.7 million deaths globally, attributed to 33 different bacterial species, including *Staphylococcus aureus*. Given their severe impact on human health, bacterial infections have become a major global health concern [1,2]. Therefore, early detection and timely treatment of bacterial contamination are paramount in safeguarding public health and preventing widespread outbreaks. In recent years, several methods for bacterial detection, such as plating and culturing [3], polymerase chain reaction [4], flow cytometry [5], fluorescence [6] and so on [7,8,9], have been developed and shown to be effective. However, these methods often come with limitations, such as lengthy processing times. Therefore, there is a pressing need for faster detection methods capable of detecting bacteria directly in the field or at the point of care, eliminating reliance on complex and time-consuming procedures.

Electrochemical biosensors based on impedance measurement offer several key advantages, including high sensitivity, rapid response, cost-effectiveness, and compatibility with miniaturized sensor platforms [10,11,12,13,14,15]. Detection of specific bacterial species can be achieved by combining the impedance technique with a suitable biorecognition element. In a typical setup, the biosensor surface is functionalized with biorecognition elements that specifically bind to the target bacteria [16]. The interaction between the immobilized biorecognition elements and the target induces changes in impedance, which can be monitored to confirm the presence and concentration of the target microorganism.

Combining electrochemical impedance-based biosensing with dielectrophoresis force (F_DEP_) has emerged as an effective approach to improve sensitivity and reduce detection time. The dielectrophoretic behavior of particles was first discovered by Reuss in clay suspensions and was subsequently investigated and formally termed dielectrophoresis by Pohl [17,18]. F_DEP_ refers to the movement of polarizable particles in a non-uniform electric field [19]. Since bacteria exhibit polarizable behavior under an applied AC field, F_DEP_ is employed to actively manipulate bacterial cells in a liquid medium, guiding them toward the sensor surface for rapid detection. The direction and magnitude of the DEP force depend on the dielectric properties of both the cells and the surrounding medium, as well as the frequency of the applied electric field. When cells are attracted toward regions of high electric field intensity, the phenomenon is termed positive DEP force (F_pDEP_), whereas movement toward lower field regions is referred to as negative DEP force (F_nDEP_) [20,21]. DEP-based bioparticle capture has been widely employed in biological applications such as cell transfer [22,23], dielectric characterization [24,25], and cell injection [26]. In addition, DEP-driven separation and sorting techniques have been extensively applied in cancer diagnostics [27], vaccine and protein purification [28,29], and cell classification [30,31], providing critical capabilities for disease diagnosis and therapeutic development. DEP has also been used for sample preparation [32], enhancement of bioassays [33,34] and molecular enrichment [35], where controlled concentration of target species significantly improves analytical performance. Collectively, these advances highlight DEP as a powerful and versatile platform for bioscience, supporting applications from cell manipulation and molecular enrichment to protein purification and disease diagnostics [36,37].

The efficiency and accuracy of the biosensor are markedly enhanced by DEP integration, which promotes targeted bioparticle accumulation at the sensing interface and facilitates effective biorecognition interaction [38,39,40]. Biorecognition elements are first immobilized on the electrode surface, and F_pDEP_ is then used to concentrate bacterial cells into regions of high electric field intensity, allowing specific binding between the surface-immobilized antibodies and their target bacteria to achieve biological selectivity [41,42]. In an alternative strategy, biorecognition elements are added directly to the bacterial suspension, promoting antibody-mediated bacterial aggregation, which increases the effective size of the target cells and enhances their F_pDEP_ response [43,44]. DEP forces can be precisely controlled by adjusting key parameters such as electric field strength, frequency, and the conductivity of the suspending medium. The non-uniform electric fields required to generate DEP forces are strongly influenced by the geometry and arrangement of electrodes within the sensing region, making electrode design a critical factor in particle trajectories, trapping efficiency, and overall device performance. Advances in microfabrication have facilitated the development of sophisticated microelectrode arrays and microscale structures, enabling precise control over electric field gradients [45]. Consequently, the configuration of the flow channel and electrode layout provides multiple degrees of freedom for selecting electrode geometries to generate maximal electric field gradients (∇E^2^) and achieve controlled and enhanced particle capture efficiency. However, existing studies on DEP-assisted bacterial sensing have predominantly employed conventional IDE geometries, with limited systematic investigation of alternative electrode designs using FEM-based modeling. In particular, quantitative evaluation of how electrode geometry affects the electric field gradient (∇E^2^), dielectrophoretic force, particle trajectories, and bacterial capture efficiency remains limited. Furthermore, operating parameters are typically selected empirically without numerical validation of the resulting DEP force spectrum. In addition, prior DEP-assisted biosensor studies rarely provide experimental comparisons between DEP-assisted (F_pDEP_ _ON) and passive (F_pDEP__OFF) detection on the same electrode geometry, leaving uncertainty about the actual contribution of dielectrophoretic enrichment to bacterial capture and electrochemical readout.

**Figure 1:**
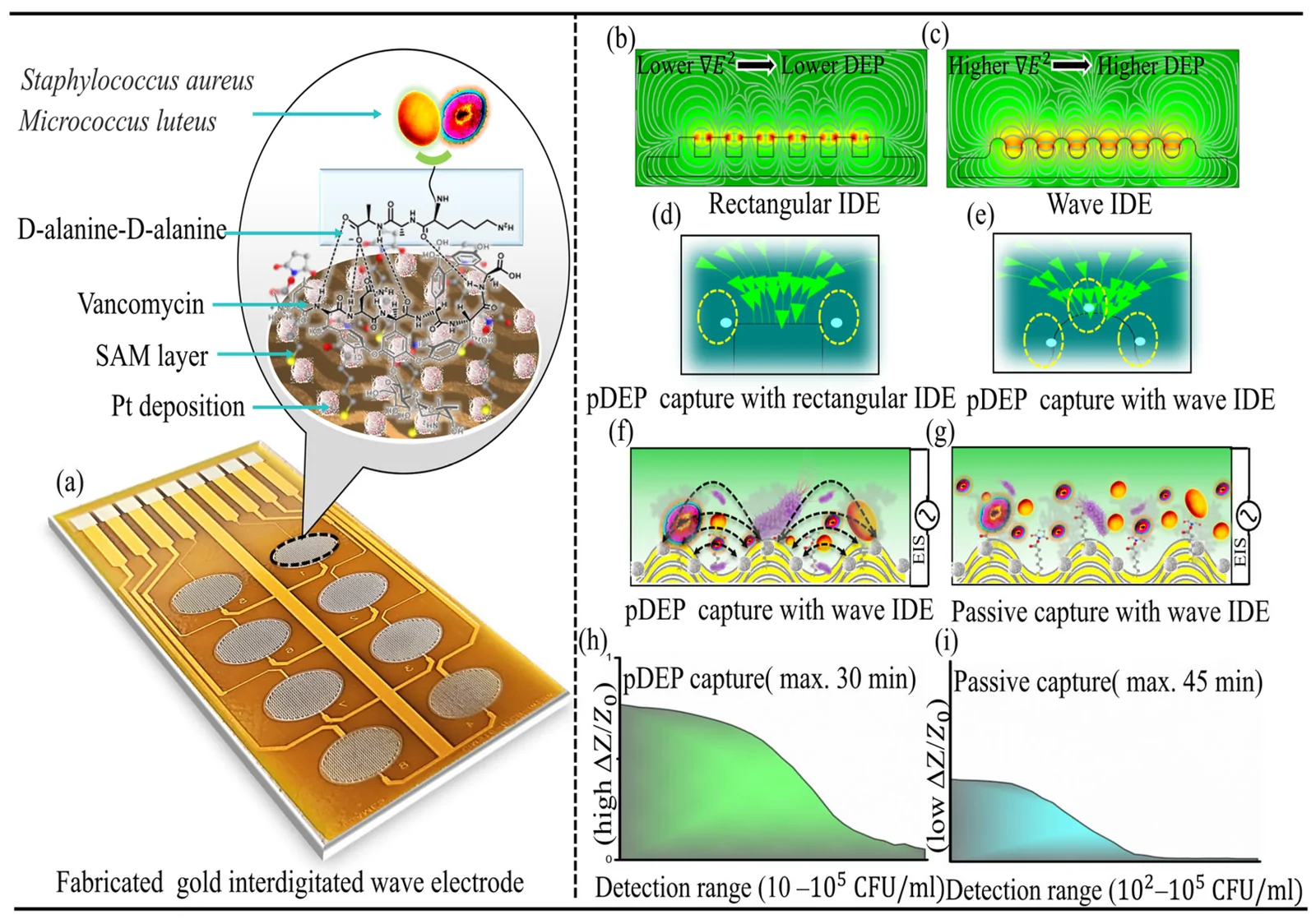
(**a**) Optical images and fabrication process of W_IDE. (**b**) COMSOL finite-element simulation of electric field distribution for rectangular R_IDE and (**c**) W_IDE. (**d**) Simulated pDEP bacterial capture regions with R_IDE and (**e**) simulated pDEP bacterial capture regions with W_IDE. (**f**) Schematic illustration of bacterial capture mechanism with pDEP-assisted (F_pDEP__ON) and (**g**) passive detection (F_pDEP__OFF). (**h**) Detection range, detection time and Δ*Z*/*Z*_0_ response of W_IDE with pDEP-assisted (F_pDEP__ON) and (**i**) passive (F_pDEP__OFF) operations.

In this study, a combined numerical–experimental framework was developed to rationally design a dielectrophoretic-assisted electrochemical biosensor with enhanced bacterial capture and detection performance. Finite-element modeling was used to solve the coupled electric field, dielectrophoretic force, and particle-transport equations, enabling quantitative comparison of different interdigitated electrode geometries in terms of bacterial capture efficiency. Guided by the simulation results, which indicated extended high-field regions, an enlarged effective capture area, and improved bacterial capture efficiency, a wave-shaped interdigitated electrode (W_IDE) biosensor was selected for fabrication. Nanomaterials have transformed viral biosensors by enabling efficient target capture and signal amplification, with graphene, carbon nanotubes, quantum dots, and metallic nanoparticles being among the most widely used platforms [46]. Accordingly, the fabricated W_IDE was functionalized with platinum nanoparticles to improve electrochemical sensitivity. The developed biosensor was then applied to pDEP-assisted electrochemical detection of Gram-positive bacteria, including *Staphylococcus aureus* and *Micrococcus luteus*, using vancomycin as the recognition element. The pDEP-assisted (F_pDEP__ON) electrochemical detection allowed rapid detection of bacteria at concentrations as low as 10 CFU/mL within 30 min. Evaluation against passive (F_pDEP__OFF) detection confirmed the substantial contribution of dielectrophoretic enrichment to both bacterial capture and electrochemical readout. The fabricated W_IDE and the comparative evaluation of pDEP-assisted and passive electrochemical detection are summarized in Figure 1. Compared with prior DEP-impedance platforms based on conventional IDE geometries, wave-shaped IDE sensors without a DEP-assisted detection mechanism, and vancomycin-based impedance sensors relying on passive capture, this work integrates FEM-guided electrode design, pDEP-driven bacterial enrichment, and vancomycin-mediated selective recognition on a single Pt-black-modified wave-shaped interdigitated electrode (W_IDE) biosensor, with direct experimental validation of the contribution of DEP enrichment.

## 2. Experimental Section

### 2.1. Reagents and Chemicals

Details of the reagents and chemicals used in this study are provided in the Supporting Information (Refer to Supplementary Section S1).

### 2.2. FEM Modeling of pDEP-Driven Bacterial Capture

**Figure 2:**
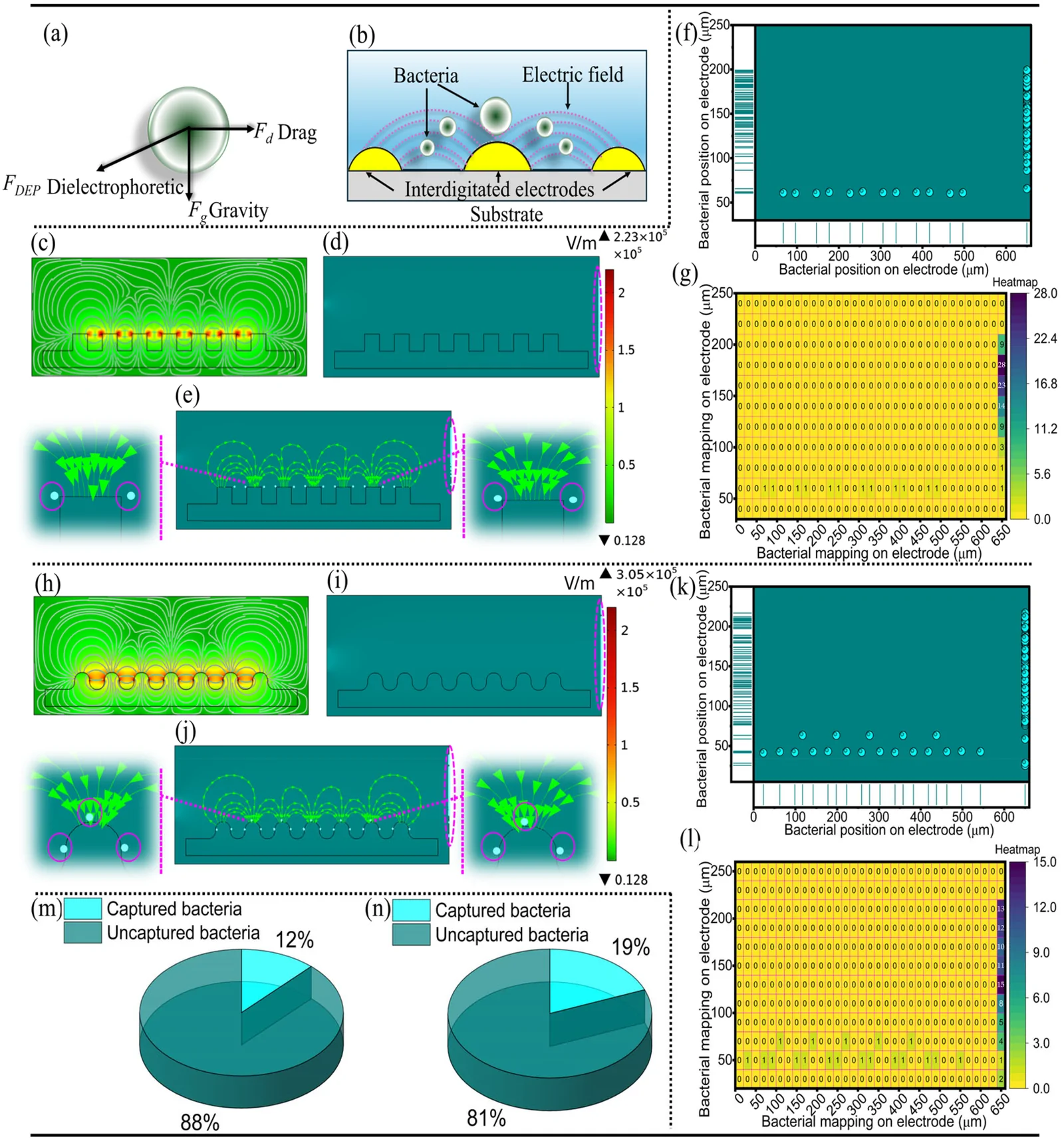
Finite-element modeling of bacterial trapping in interdigitated electrodes. (**a**) Schematic illustration of the forces acting on a *S. aureus* cell. (**b**) Schematic representation of *S. aureus* subjected to dielectrophoretic forces in the electrolyte over an interdigitated electrode. (**c**) COMSOL-simulated electric field distribution for the rectangular interdigitated electrode (R_IDE). (**d**,**e**) COMSOL-simulated bacterial capture on the R_IDE in the absence (F_pDEP__OFF) and presence (F_pDEP__ON) of positive dielectrophoresis, respectively. (**f**) Scattering plot showing the spatial positions of bacteria on the R_IDE surface. (**g**) Corresponding bacterial density (mapping) plot for the R_IDE. (**h**) COMSOL-simulated electric field distribution for the wave-shaped interdigitated electrode (W_IDE). (**i**,**j**) COMSOL-simulated bacterial capture on the W_IDE in the absence (F_pDEP__OFF) and presence (F_pDEP__ON) of positive dielectrophoresis, respectively. (**k**) Scattering plot of bacterial positions on the W_IDE surface. (**l**) Corresponding bacterial density (mapping) plot for the W_IDE (**m**,**n**). Quantitative comparison of bacterial capture efficiency for the R_IDE and W_IDE under positive dielectrophoresis (F_pDEP_), respectively.

To evaluate the influence of electrode geometry on DEP-mediated bacterial trapping, a finite-element (FEM) model was developed in COMSOL Multiphysics 5.6. The model comprised two 2D electrode configurations, the interdigitated rectangular electrode (R_IDE) and interdigitated wave electrode (W_IDE), which were used to comparatively assess bacterial trapping behavior and capture efficiency. It was assumed that when a droplet containing *S. aureus* cells is applied to the biosensor, the cells move freely in the solution and are captured upon entering the active sensing region of the electrodes. The simulations were performed by considering only the dielectrophoretic force (F_DEP_) and the Stokes drag force (F_d_), while gravitational (F_g_) and Brownian forces (F_b_) were neglected. Schematics of the computational model used for the simulation are shown in Figure 2a,b, and the physical properties used in the simulation are summarized in Table 1. The model was assigned the physical properties of phosphate-buffered saline (PBS) as the medium, and the bacterial cells those of *Staphylococcus aureus* spheres.

The numerical analysis in COMSOL for the electric field distribution was performed using the electric currents physics interface, governed by the following equations:

(1) ∇⋅J=Q

(2) $$J=\sigma E+j\omega D+J_{e}$$

(3) E=−∇V

where J, Q, σ, D, ω, V are the current density, charge density, electrical conductivity, electric displacement, angular frequency and electric potential, respectively. Electrical insulating boundary conditions were set on all external boundaries, except for the electrode surfaces. The electrodes were assigned voltage boundary conditions, and the electric current interface was solved in the frequency domain (1 Hz to 100 kHz) to determine the spatial distribution of the electric field surrounding the electrodes.

The particle tracing interface was coupled with the electric current and creeping flow interfaces to compute the forces acting on the bacterial cells (*Staphylococcus aureus*), including the dielectrophoretic (F_DEP_) and Stokes drag (F_d_) forces. For spherical particles of radius $r_{p}$, the F_DEP_ is computed as:

(4) $$F_{DEP}=2\pi r_{p}^{3}\varepsilon_{m}Re\left[K\left(\omega \right)\right]\nabla |E^{2}$$

where $r_{p}$ denotes the particle radius, $\varepsilon_{m}$ represents the dielectric constant of the surrounding medium, ∇ is the vector differential operator, and Re[K(ω)] corresponds to the real component of the Clausius–Mossotti factor, which is defined in Equation (5):

(5) $$K\left(\omega \right)=\frac{\varepsilon_{p}^{*}-\varepsilon_{m}^{*}}{\varepsilon_{p}^{*}+2\varepsilon_{m}^{*}}\;\;$$

Here, $\varepsilon_{p}^{*}$ and $\varepsilon_{m}^{*}$ are the complex permittivities of the particle and medium and are represented by Equation (6):

(6) $${\varepsilon_{p}}^{*}=\varepsilon_{p}-\frac{j\sigma_{p}}{\omega },\;{\varepsilon_{m}}^{*}=\varepsilon_{m}-\frac{j\sigma_{m}}{\omega }$$

Since the complex permittivity in Equation (6) contains the conductivity term σ/ω, the Clausius–Mossotti factor in Equation (5) depends explicitly on the medium conductivity $\sigma_{m}$. Decreasing $\sigma_{m}$ reduces the imaginary part of $\varepsilon_{m}^{*}$ and increases $Re\left[K\left(\omega \right)\right]$, thereby strengthening the DEP force and enhancing bacterial trapping. Conversely, increasing $\sigma_{m}$ increases the imaginary part of $\varepsilon_{m}^{*}$, which reduces $Re\left[K\left(\omega \right)\right]$, weakens the pDEP force, and can shift the crossover frequency at sufficiently high conductivity. Furthermore, Re[K(ω)] depends on the frequency of the applied AC field; the magnitude and direction of the F_DEP_ are frequency dependent. A positive value of Re[K(ω)] results in positive F_DEP_, attracting particles toward regions of high electric field intensity, while a negative value leads to negative F_DEP,_ repelling particles from such regions. The particle motion is further influenced by the Stokes drag force, calculated as:

(7) $$F_{d}=3µ\pi d_{p}\left(u_{m}-v_{p}\right)$$

where $u_{m}$ is the velocity of the medium, $d_{p}$ refers to the diameter of the bacterium, µ represents the viscosity of the medium, and $v_{p}$ corresponds to the velocity of the particles. The particle trajectories were determined through a time-dependent particle tracing simulation to evaluate how the combined effects of the electric field distribution and dielectrophoretic (DEP) and Stokes drag forces influence bacterial transport and capture within the interdigitated electrodes.

The simulation results for the interdigitated rectangular electrode (R_IDE) and interdigitated wave electrode (W_IDE) are summarized in Figure 2. For the R_IDE configuration, the electric field distribution was calculated using the electric current interface, as shown in Figure 2c. Particle tracing simulations were subsequently performed both in the presence and absence of the F_DEP_, as shown in Figure 2d,e. In the presence of F_DEP_, bacterial cells were preferentially attached to the electrode edges, where the electric field intensity is highest, as depicted in Figure 2e. Conversely, in the absence of F_DEP_, bacterial cells remained randomly distributed and exhibited no attachment to the electrode surface (Figure 2d). Figure 2f shows a scatter plot of 100 bacterial cells, with 12 cells captured at the electrode edges and the remaining cells freely distributed in other regions. Similarly, Figure 2g shows the corresponding bacterial mapping plot, which quantifies the number of bacterial cells at each position on the electrode surface and surrounding area, highlighting the preferential accumulation at the high-field edge regions. Next, the W_IDE configuration was investigated for the electric field distribution using the electric currents interface, as shown in Figure 2h. Particle tracing simulations in the presence of F_DEP_ revealed that bacterial cells were captured not only at the edges, but also in the mid-wave region of the wave-shaped electrodes, as shown in Figure 2j. However, without F_DEP_, the cells followed unperturbed paths and remained dispersed across the domain, showing no interaction with the electrode surface as shown in Figure 2i. Figure 2k illustrates the spatial distribution of 100 bacterial cells, showing that 19 cells accumulate at the electrode surface, whereas the rest are dispersed across the domain. Similarly, Figure 2l depicts the bacterial density map for the W_IDE configuration, illustrating the number of cells across both the electrode surface and adjacent regions.

The capture efficiency of both electrode geometries (R_IDE and W_IDE) is quantified in Figure 2m,n. The R_IDE achieved a bacterial capture efficiency of 12%, whereas the W_IDE reached 19%, representing a notable improvement. This enhancement is attributed to the wave-shaped geometry, which produces additional high-electric-field regions across the curved regions of the wave electrodes, extending the effective capture area and allowing more bacterial cells to be captured compared to the conventional rectangular electrode, where high field intensity is largely confined to the edges. Based on the enhanced capture efficiency predicted by simulations, the wave-shaped interdigitated electrode was chosen for fabrication and subsequent experimental studies.

### 2.3. Fabrication of W_IDE Sensor Array and Multichannel Electrochemical System

**Figure 3:**
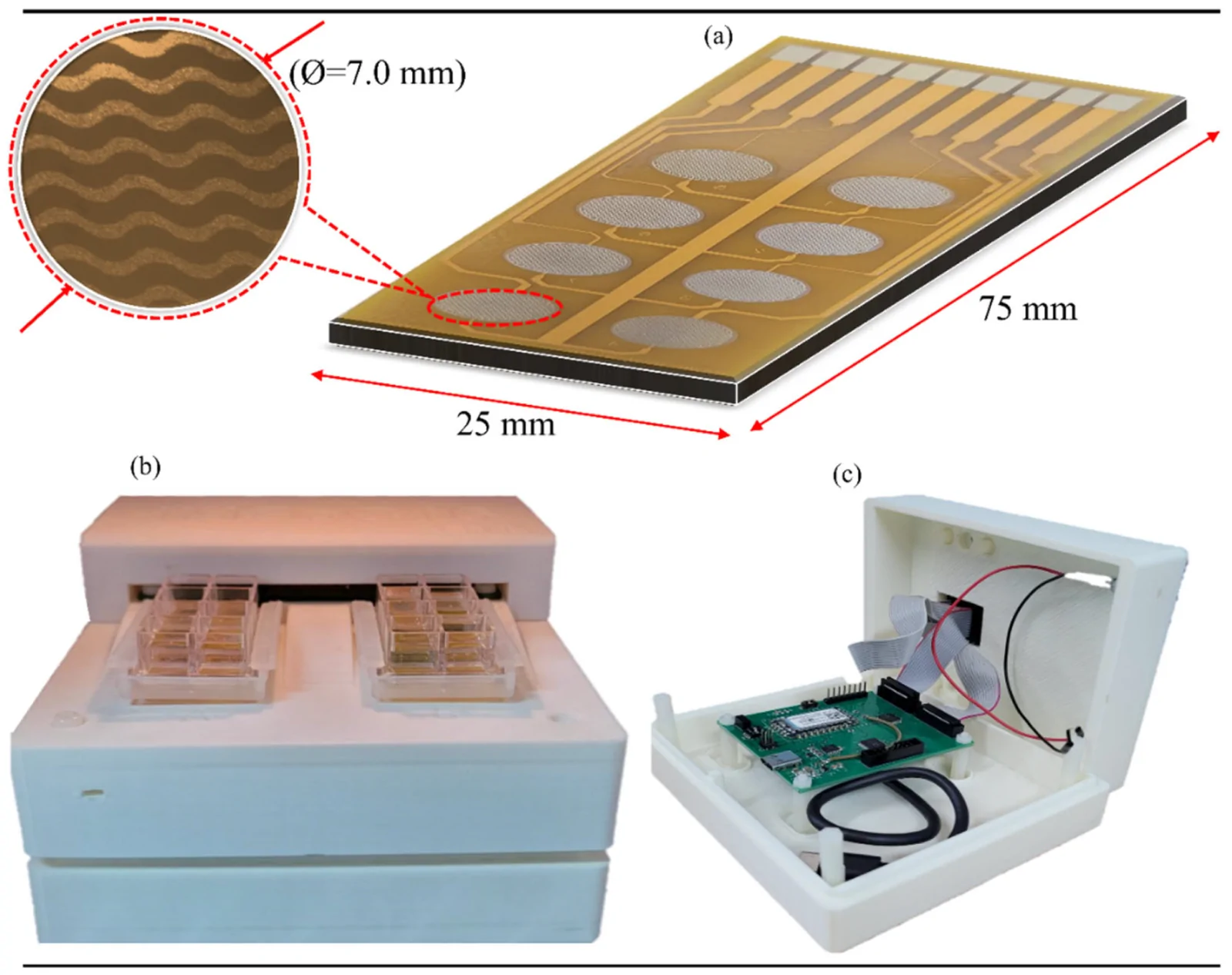
Optical images of the DEP-assisted electrochemical biosensing platform. (**a**) Fabricated 8-channel W_IDE array with common counter electrode. (**b**) Custom 3D-printed holder enabling controlled bacterial incubation. (**c**) Custom-designed 16-channel electrochemical measurement unit based on PCB technology, integrating an EmStat Pico potentiostat.

The interdigitated wave-shaped electrode (W_IDE) was fabricated by CELLAMES (Seongnam, Republic of Korea) on a PCB substrate, as shown in Figure 3a. The sensor array consists of eight working/sensing electrodes with a shared counter electrode. Nickel (3 µm) and gold (0.8 µm) were sequentially deposited on a 1 mm thick rigid FR-4 PCB substrate using electron-beam evaporation. The working and counter electrodes featured widths and inter-electrode gaps of 100 µm. A 25 mm × 75 mm PCB substrate accommodated the W_IDEs, with each array featuring an active sensing area 7.0 mm in diameter. For bacterial incubation and subsequent electrochemical measurements, a custom-designed holder was 3D-printed, as shown in Figure 3b. The holder accommodates two W_IDEs sensor arrays, enabling simultaneous processing of 16 sensing electrodes. It incorporates individual wells positioned directly above each sensing site to provide isolated microenvironments for controlled sample loading, incubation, and washing. The electrochemical measurement unit was developed by integrating a commercial EmStat Pico potentiostat (EmStat Pico MUX16, PalmSens BV, Houten, The Netherlands) onto a custom-designed printed circuit board (PCB) engineered to support multiplexed electrochemical measurements across 16 channels, as shown in Figure 3c. The EmStat Pico core is interfaced with two 16-channel multiplexers, a USB-C communication module, and an on-board 3.3 V power regulation circuit, enabling sequential addressing of 16 sensing electrodes. A complete list of the electrochemical system components, along with the detailed board design, is provided in Table S1 and Figure S1 (Refer to Supplementary Data). The measurement system is housed within the designed 3D-printed holder (Figure 3c), ensuring reliable electrical interfacing with the sensor arrays and providing a compact, portable platform for real-time, multichannel electrochemical analysis.

### 2.4. Fabrication of the W_IDE Biosensor

The bare W_IDE array was cleaned with ethanol and deionized water to remove surface impurities, dried with nitrogen gas, and subsequently treated with oxygen plasma. To enhance surface activity, the W_IDE was modified by electrodeposition of Pt-black, forming a porous nanostructure. Electrodeposition was performed in the sensing area using a three-electrode configuration, with a solution containing hydrogen hexachloroplatinate (IV) hexahydrate (2.5%), lead diacetate trihydrate (0.05%), and HCl (0.01 M). A constant current density of −2.5 mA cm^−2^ was applied for deposition times of 120 s, 240 s, and 400 s to systematically investigate the evolution of Pt-black morphology and its effect on the electrode’s electroactive surface area and charge-transfer performance. For the optimum deposition time of 400 s, the corresponding Pt-black mass loading was calculated to be 505 μg cm^−2^. The morphology and surface characteristics of the electrodeposited Pt-black were examined using scanning electron microscopy (SEM) (Hitachi SU8600, Tokyo, Japan) and atomic force microscopy (AFM) (XE-100, Park Systems, Suwon, Republic of Korea). The developed Pt-black-modified W_IDE was washed and then immersed in a 6-mercaptohexanoic acid solution (100 mM) for 12 h, followed by EDC/NHS activation for 15 min at room temperature. The use of 6-mercaptohexanoic acid (100 mM) was based on previously reported SAM functionalization protocols [52,53] and facilitated surface functionalization of the highly porous, nanostructured morphology of the Pt-black-modified electrode, providing stable and reproducible SAMs for our electrode geometry and incubation time. Finally, the prepared electrode was incubated with vancomycin solution (2 mg/mL) for 2 h and subsequently treated with 0.5% BSA to block nonspecific binding sites, completing the biosensor fabrication process.

### 2.5. Preparation of Bacterial Cultures

The bacterial cultures of *Staphylococcus aureus* and *Micrococcus luteus* were initially grown in a liquid growth medium under optimal incubation conditions until reaching the exponential growth phase. Following incubation, 100 μL of each culture was collected, and the optical density (OD) at 600 nm was measured using a spectrophotometer (OPTIZEN POP, K LAB Co., Ltd., Daejeon, Republic of Korea) to estimate bacterial concentration. The cultures were then centrifuged and washed with phosphate-buffered saline (PBS) to remove residual growth medium, and the bacterial pellets were resuspended in PBS. Serial dilutions were subsequently performed in PBS to achieve the desired working concentrations ranging from 10 to 10^5^ CFU/mL for electrochemical detection experiments.

### 2.6. Electrochemical Measurements

Electrochemical impedance spectroscopy (EIS) was used to evaluate bacterial detection under both pDEP-assisted (F_pDEP__ON) and passive (F_pDEP__OFF) conditions. For each measurement, 700 μL of bacterial suspensions (10–10^5^ CFU/mL) were introduced onto vancomycin-functionalized wave-shaped interdigitated electrodes (W_IDEs). In pDEP-assisted measurements, an AC electric field (100 kHz, 10 Vpp) was applied for 30 min to actively concentrate bacteria at the electrode surface, followed by impedance recording. Passive detection was performed without an applied electric field, using a 45 min incubation period before measurement. The solution temperature variation during the enrichment period was confirmed to be less than 1.3 °C, ruling out electrothermally induced fluid motion and evaporation as contributors to bacterial enrichment. The duration of pDEP application (30 min) and passive incubation (45 min) were selected based on time-course optimization experiments. The W_IDE array was mounted in a custom-designed 3D-printed holder and electrically interfaced with our portable electrochemical measurement system based on an EmStat Pico potentiostat. Optical images of the experimental setup are shown in Figure S2 (Refer to Supplementary Data). EIS measurements for bacterial detection were conducted in PBS over a frequency range of 1 Hz to 100 kHz, while Nyquist and Bode plot analyses for electrode characterization were performed in 5 mM [Fe (CN)_6_]^3−^/^4−^. All measurements were repeated at least three times to ensure reproducibility.

## 3. Results and Discussion

### 3.1. Morphological and Electrochemical Characterization of Pt-Black-Modified W_IDE

**Figure 4:**
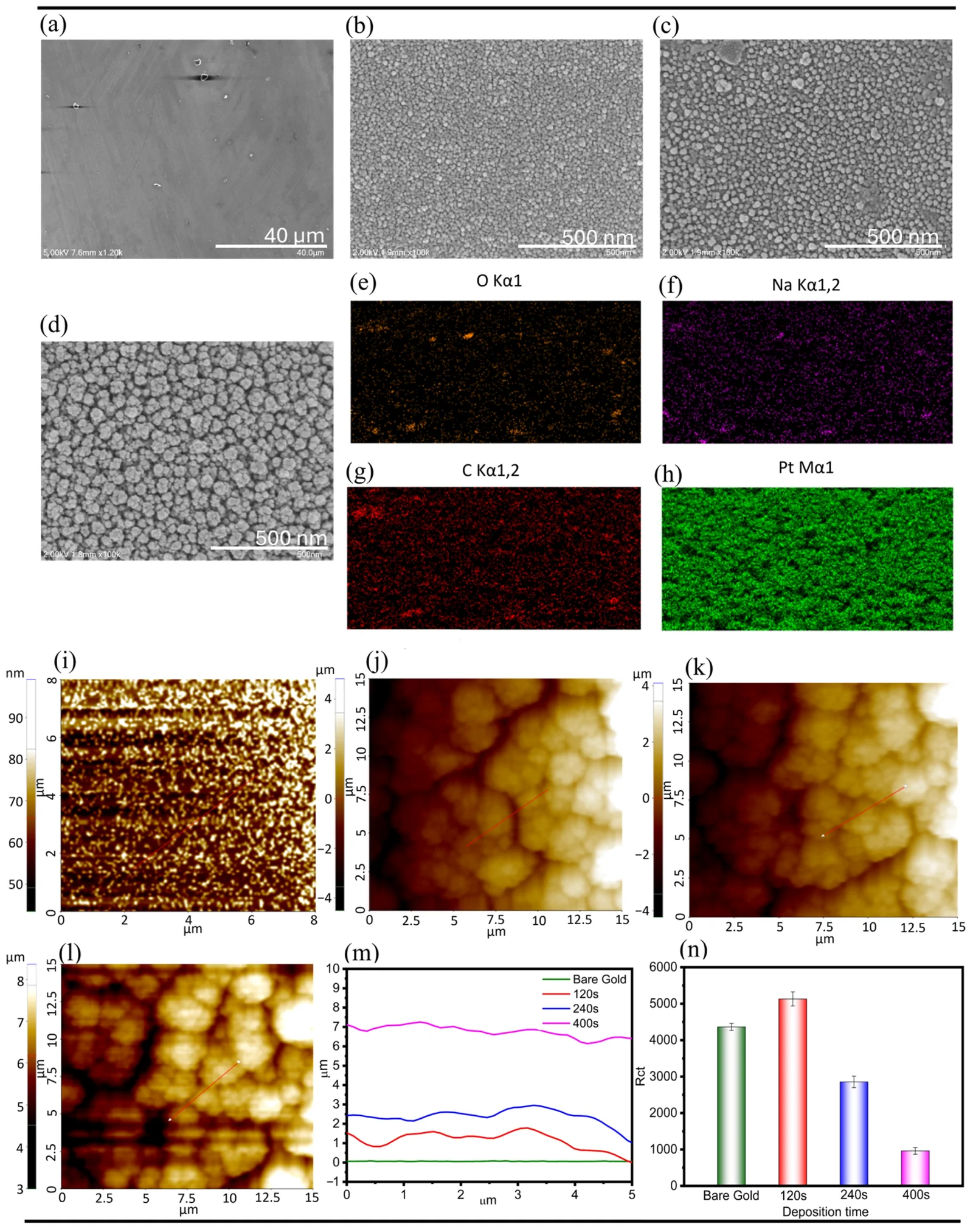
Morphological and electrochemical characterization of Pt-black electrodeposition on the W_IDE surface. (**a**) SEM image of the bare gold W_IDE; (**b**–**d**) SEM images showing Pt-black growth after electrodeposition for 120 s, 240 s, and 400 s, respectively; (**e**–**h**) EDS mapping confirming uniform Pt distribution on the W_IDE surface following deposition; (**i**) AFM surface topologies of the bare W_IDE; and (**j**–**l**) AFM surface topologies of Pt-black-modified W_IDE after electrodeposition for 120 s, 240 s, and 400 s, respectively. The red lines in the AFM images indicate the locations of the line profiles used for quantitative surface height analysis. (**m**) Quantitative AFM surface roughness analysis showing increased height variation with longer deposition times. (**n**) EIS measurements of Pt-black-modified W_IDE at different deposition times, indicating enhanced charge-transfer kinetics at 400 s.

Pt-black was electrodeposited onto the wave-shaped gold electrodes (W_IDE) to enhance their electroactive surface and overall biosensor performance. Scanning electron microscopy (SEM) was used to examine the surface morphology and evaluate the effect of deposition time on Pt-black growth. The morphology of the bare gold electrode is shown in Figure 4a, while Figure 4b–d illustrate the progressive Pt-black deposition growth at 120 s, 240 s, and 400 s, respectively. As shown in Figure 4b–d, increasing the deposition time leads to the Pt-black transition from sparse nucleation at 120 s (Figure 4b) to partially merged clusters at 240 s (Figure 4c), and ultimately to a densely packed cauliflower-like structure at 400 s (Figure 4d), a morphology known to provide a high electroactive surface area and improved charge-transfer characteristics [54]. The electrodes were further inspected and subjected to an electrical continuity test after 400 s of Pt-black electrodeposition, and no electrical short circuit or unintended conductive bridging across the inter-electrode gaps was detected. EDS mapping (Figure 4e–h) confirms the successful deposition of Pt-black, showing a clear and uniform Pt distribution on the gold W_IDE surface. AFM imaging (Figure 4i–l) was also carried out to assess the evolution of the surface morphology with different deposition times. The AFM topography reveals a progressive increase in surface height variation as the deposition time increases, indicating that the surface becomes more structurally developed at 400 s compared to the shorter deposition conditions, as illustrated in Figure 4m. Electrochemical impedance spectroscopy (Figure 4n) further supports the morphological findings, showing that the electrode with the deposition time of 400 s exhibits the lowest charge-transfer resistance (Rct), corresponding to an approximate 77% decrease relative to the bare electrode. This substantial decrease indicates significantly enhanced electron-transfer kinetics resulting from the densely packed, cauliflower-like Pt-black structure. In contrast, shorter deposition times (120 s) result in incomplete Pt coverage, leading to higher charge-transfer resistance. At this early stage of deposition, Pt forms isolated and discontinuous islands on the Au substrate, thereby limiting faradaic electron transfer to a fraction of the electrode surface. This reduced effective electrochemically active area lowers the exchange current density of the composite interface, resulting in a higher Rct. Similarly, excessive deposition can induce nanoparticle agglomeration, reducing electrochemical efficiency. These findings highlight the critical role of controlled Pt-black growth for developing electrode surfaces with enhanced electron-transfer properties and improved biosensor performance.

### 3.2. Electrochemical Detection of Bacteria Using Passive and DEP-Assisted Approaches

**Figure 5:**
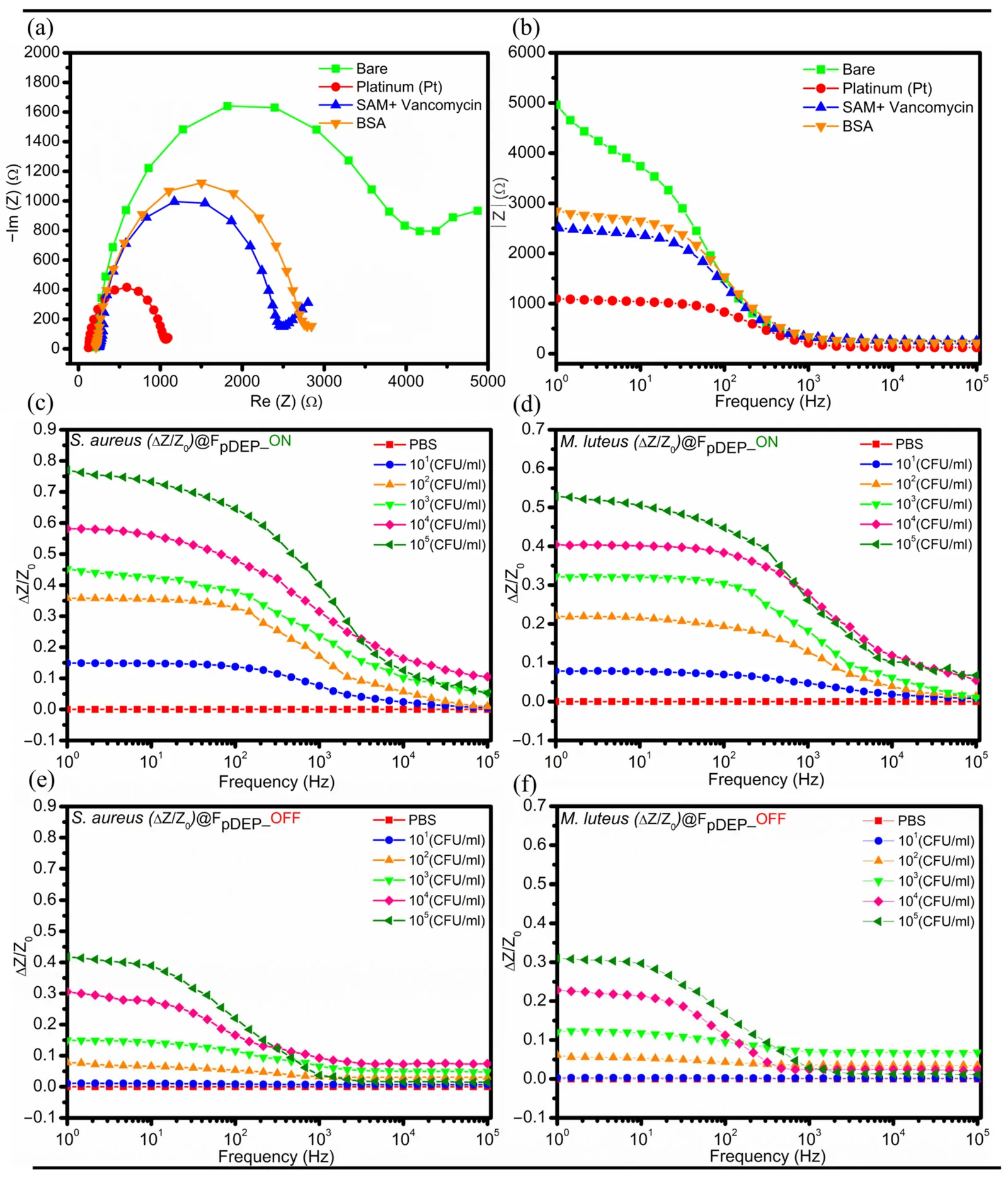
Electrochemical characterization and bacterial detection performance of the W_IDE biosensor under pDEP-assisted (F_pDEP__ON) and passive (F_pDEP__OFF) bacterial detection. (**a**) Nyquist plot showing stepwise surface functionalization of the W_IDE (bare Au, Pt-black, SAM, vancomycin, and BSA) in 5 mM [Fe(CN)_6_]^3−^/^4−^. (**b**) Corresponding Bode magnitude plots after each modification step. (**c**,**d**) Impedance response (Δ*Z*/*Z*_0_) of *S. aureus* and *M. luteus* over a concentration range of 10–10^5^ CFU/mL under pDEP-assisted (F_pDEP__ON) detection (**e**,**f**) Impedance response (Δ*Z*/*Z*_0_) of *S. aureus* and *M. luteus* over a concentration range of 10–10^5^ CFU/mL under passive (F_pDEP__OFF) detection.

Prior to bacterial detection, EIS was used to assess the stepwise functionalization and detection capability of the biosensor in 5 mM [Fe (CN)_6_]^3−^/^4−^ (1 Hz–100 kHz). Figure 5a,b illustrates how each surface modification step affects the electrode’s charge transfer resistance (Rct) and impedance response, as reflected in the Nyquist and Bode plots. As shown in Figure 5a, the bare gold W_IDE showed a high Rct; however, the Pt-black deposition significantly reduced this value due to enhanced conductivity and increased electroactive surface area. Upon SAM modification, Rct increased, reflecting the reduced electron-transfer kinetics caused by the insulating monolayer. The subsequent attachment of vancomycin produced a further increase in Rct, confirming successful attachment. Finally, BSA blocking resulted in an additional rise in Rct indicative of surface passivation through protein adsorption. Similar trends were observed in the Bode plot (Figure 5b), where the impedance initially decreased after Pt-black deposition and then increased sequentially with SAM, vancomycin, and BSA modification.

Next, the biosensor’s performance for detecting Gram-positive bacteria (*S. aureus* and *M. luteus*) was assessed in the presence and absence of positive dielectrophoresis (pDEP). The pDEP forces enhance bacterial accumulation at the sensor surface by using non-uniform electric fields that drive highly polarizable cells toward regions of high field intensity [55]. Bacterial suspensions of *S. aureus* and *M. luteus,* prepared in 0.1× PBS at concentrations of 10–10^5^ CFU/mL, were introduced onto the W_IDE biosensor surface. For pDEP-assisted experiments, an AC electric field (100 kHz, 10 Vpp) was applied for 30 min to induce positive dielectrophoresis, facilitating rapid accumulation of cells at the sensing surface while minimizing joule heating, preventing cell damage and avoiding undesired reactions [56]. For comparison, control measurements were performed under identical conditions in the absence of pDEP, enabling the evaluation of passive bacterial capture. The corresponding impedance responses for pDEP-assisted and passive bacterial binding are presented in Figure 5c,d and Figure 5e,f, respectively. For the pDEP-assisted experiments, the impedance was recorded immediately after applying the AC field for 30 min, while passive binding was evaluated following a 45 min incubation period in the absence of an applied field. The impedance responses were calculated as follows:

(8) $${\left(\frac{\Delta Z}{Z_{0}}\right)}_{pDEP}=\;\frac{Z_{pDEP}-Z_{0}}{Z_{0}}$$

(9) $${\left(\frac{\Delta Z}{Z_{0}}\right)}_{passive}=\frac{Z_{passive}-Z_{0}}{Z_{0}}$$

where $Z_{0}$ represents the baseline impedance of the biosensor prior to bacterial exposure, $Z_{pDEP}$ corresponds to the impedance measured following pDEP-assisted bacterial binding, and $Z_{passive}$ is the impedance measured in the absence of an applied pDEP electric field.

As shown in Figure 5c,d, *S. aureus* and *M. luteus* produced measurable impedance responses across the tested concentration range of 10 to 10^5^ CFU/mL under pDEP-assisted conditions, with $\frac{\Delta Z}{Z_{0}}$ increasing linearly with bacterial concentration. In contrast, under passive conditions, $\frac{\Delta Z}{Z_{0}}$ values were significantly lower, and the lowest concentration of 10 CFU/mL was indistinguishable from the bacterial-free medium, as shown in Figure 5e,f. It should be noted that the weak response observed experimentally under passive conditions is attributed to nonspecific bacterial adsorption during static incubation, while no attachment is predicted in COMSOL simulations without pDEP due to the absence of passive adsorption mechanisms in the model. These results confirm that pDEP significantly enhances bacterial accumulation at the sensor surface and improves measurement sensitivity at very low concentrations.

**Figure 6:**
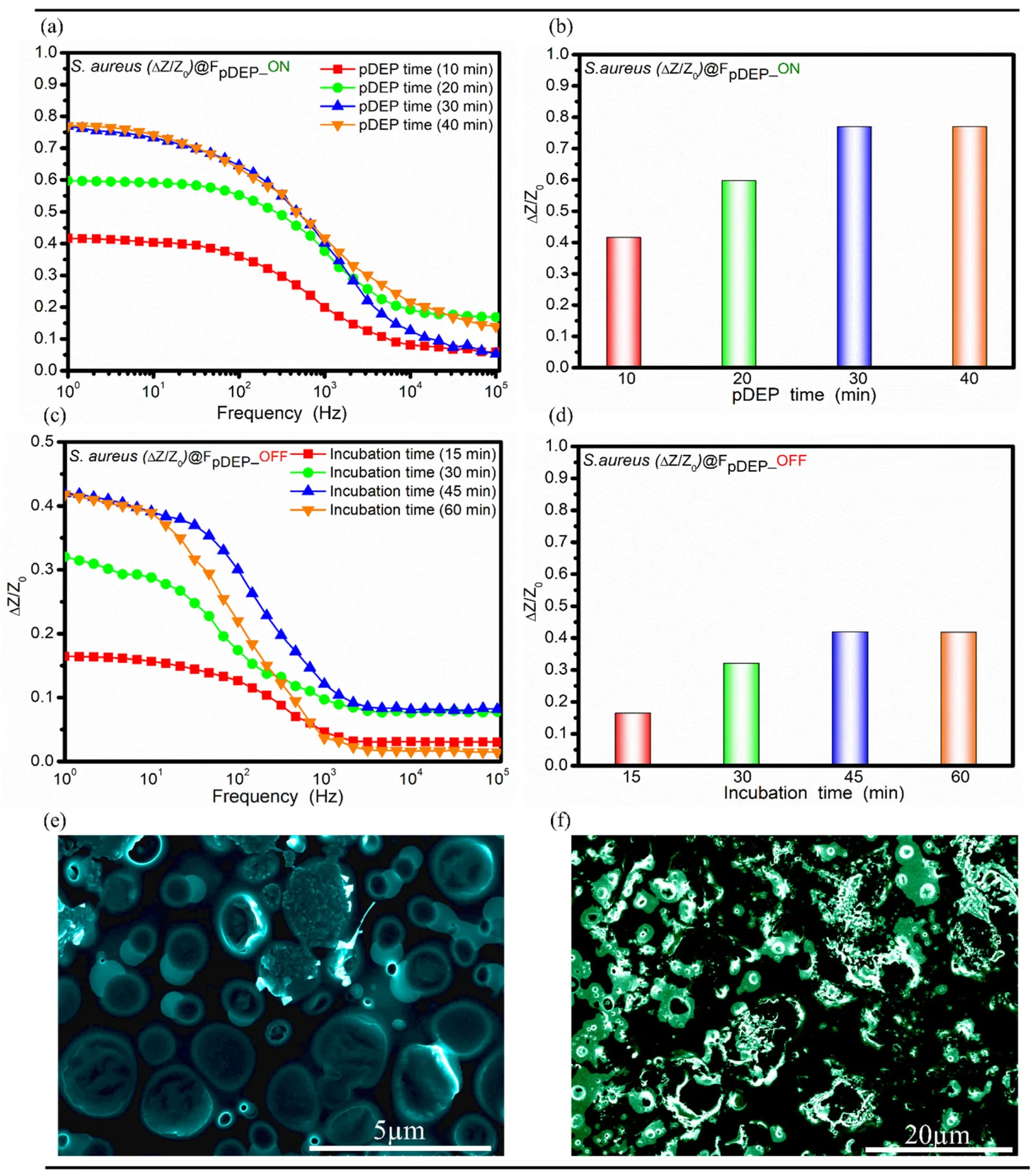
Optimization of bacterial detection time using Δ*Z*/*Z*_0_ measurements for *S. aureus* (10^5^ CFU/mL); (**a**) Δ*Z*/*Z*_0_ spectra measured at different DEP application times; (**b**) corresponding bar plots of Δ*Z*/*Z*_0_ values for quantitative comparison; (**c**) Δ*Z*/*Z*_0_ responses obtained at different passive incubation times; and (**d**) corresponding bar plots of Δ*Z*/*Z*_0_ values. Scanning electron microscopy (SEM) images of (**e**) *Staphylococcus aureus* and (**f**) *Micrococcus luteus*.

The DEP enrichment time and passive incubation period were systematically optimized by monitoring the normalized impedance response (Δ*Z*/*Z*_0_) at different time intervals, as shown in Figure 6a,d. The impedance (Δ*Z*/*Z*_0_) signal increased with DEP enrichment time before reaching a plateau at 30 min, indicating sufficient bacterial accumulation at the sensing surface, as shown in Figure 6a,b. Similarly, in the case of passive incubation, the impedance response (Δ*Z*/*Z*_0_) gradually increased and stabilized after 45 min, suggesting that longer incubation provided no significant improvement in bacterial capture, as illustrated in Figure 6c,d. Therefore, 30 min and 45 min were selected as the optimal enrichment and incubation times for DEP-assisted and passive detection, respectively.

In this study, 0.1× PBS (σ ≈ 0.16 S m^−1^) was used to balance pDEP performance and physiological relevance. This conductivity maintained a positive Re[K(ω)] and effective pDEP forces at the selected frequency. However, conductivity-dependent variations in pDEP force may influence bacterial enrichment and detection time; therefore, the 30 min enrichment time is specific to this condition. Future studies will systematically evaluate different conductivities to optimize enrichment and detection time.

To visually confirm bacterial capture following electrochemical detection, scanning electron microscopy (SEM) was used to image bacterial cells captured on the W_IDE surface, as shown in Figure 6e,f. The corresponding bacterial fixation and SEM preparation procedures are detailed in the Supporting Information (Refer to Supplementary Section S2).

**Figure 7:**
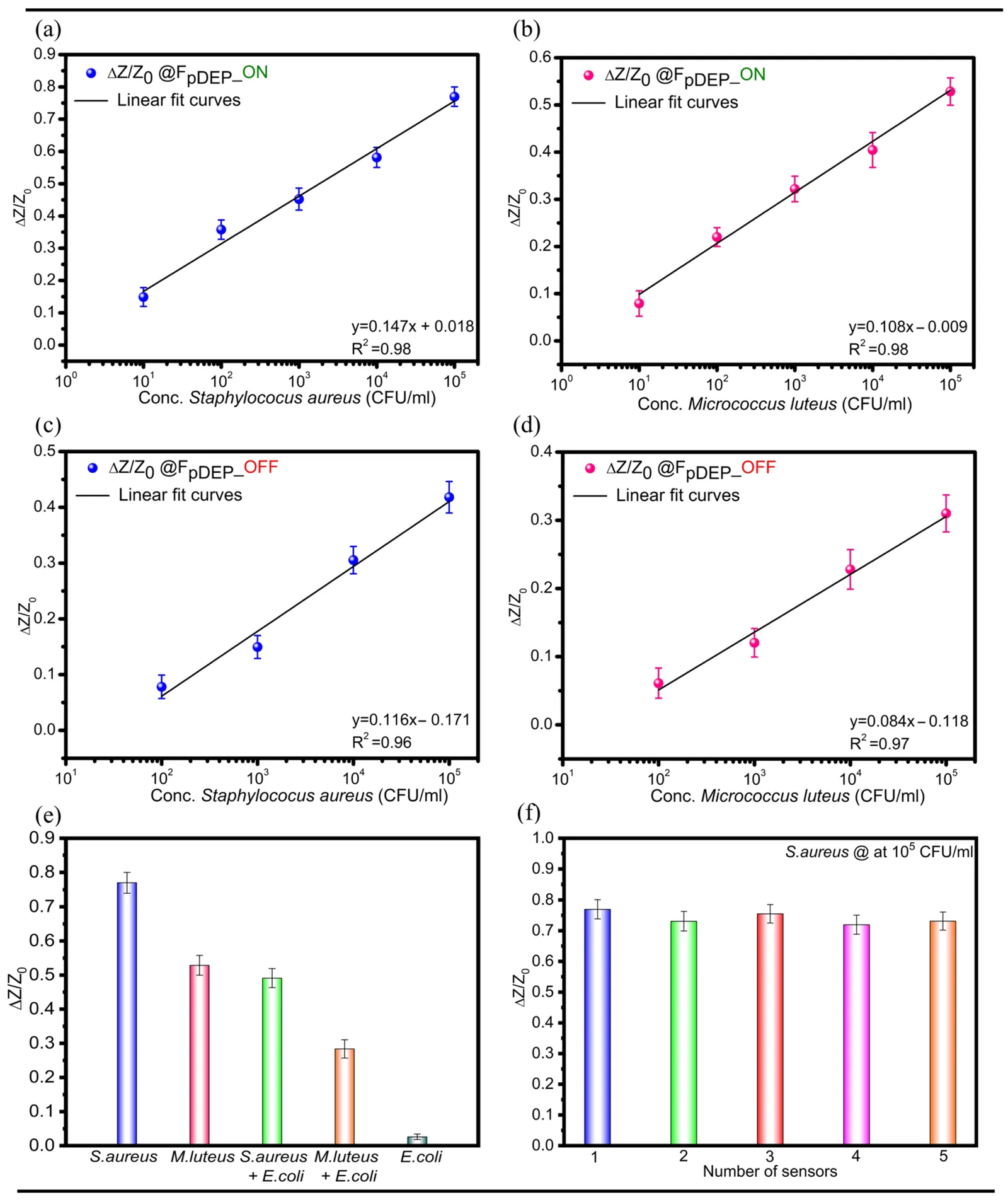
Calibration curves for electrochemical impedance-based bacterial detection under pDEP-assisted (F_pDEP__ON) and passive (F_pDEP__OFF) conditions at 1 Hz as a function of different bacterial concentrations. (**a**,**b**) Δ*Z*/*Z*_0_ values measured at bacterial concentration (10–10^5^ CFU/mL) for *S. aureus* and *M. luteus* under pDEP-assisted conditions. (**c**,**d**) Δ*Z*/*Z*_0_ values obtained at bacterial concentration (10^2^–10^5^ CFU/mL) for *S. aureus* and *M. luteus* under passive detection conditions. Δ*Z*/*Z*_0_ responses of the pDEP-assisted W_IDE biosensor at f = 1 Hz showing (**e**) specificity using different bacterial species at 10^5^ CFU/mL and (**f**) reproducibility at 10^5^ CFU/mL. Data are presented as mean values (n = 3), with error bars indicating the standard deviation.

To quantify bacterial detection performance under both pDEP-assisted (FpDEP_ON) and passive conditions (FpDEP_OFF), calibration curves were constructed by plotting $\frac{\Delta Z}{Z_{0}}$ values measured at 1 Hz against bacterial concentration, as illustrated in Figure 7a–f. For pDEP-assisted conditions, linear relationships were obtained for *S. aureus* and *M. luteus* over a wide concentration range of 10–10^5^ CFU/mL, yielding regression equations of (y = 0.147x + 0.018; R^2^ = 0.98) and (y = 0.108x − 0.009; R^2^ = 0.98), respectively, as shown in Figure 7a,b. In comparison, passive detection performed in the absence of pDEP exhibited linear behavior over a narrower concentration range of 10^2^–10^5^ CFU/mL, with regression equations of (y = 0.116x − 0.171; R^2^ = 0.96) and (y = 0.084x − 0.118; R^2^ = 0.97) for *S. aureus* and *M. luteus*, respectively, as illustrated in Figure 7c,d. The extended linear range and higher regression coefficients obtained under pDEP-assisted conditions confirm the role of dielectrophoretic forces in improving signal linearity and sensitivity.

### 3.3. Selectivity and Specificity Analysis

Furthermore, the specificity of our developed W_IDE electrochemical biosensor was evaluated by testing the representative Gram-positive bacteria *S. aureus* and *M. luteus*, the Gram-negative bacterium *E. coli*, and mixed suspensions of *S. aureus* + *E. coli* and *M. luteus* + *E. coli* under identical pDEP-assisted conditions. All bacterial suspensions were prepared at the same concentration (10^5^ CFU/mL) and subjected to positive dielectrophoretic forces prior to electrochemical measurement. Despite the application of DEP, which can physically transport different bacterial species toward the electrode surface, a pronounced impedance response was observed exclusively for Gram-positive bacteria. In contrast, *E. coli* produced a negligible signal, indicating minimal nonspecific interaction, as depicted in Figure 7e. This selective response arises from the specific binding of vancomycin to terminal D-alanine–D-alanine residues in the peptidoglycan layer of Gram-positive bacteria [57], whereas the outer membrane of Gram-negative bacteria inhibits vancomycin access and binding. The mixed bacterial suspension also exhibited a measurable impedance response, although its magnitude was lower than that of Gram-positive bacteria alone. This reduced response can be attributed to the physical presence of non-target *E. coli* cells. Based on established dielectrophoresis (DEP) theory and the applied parameters (100 kHz, 10 Vpp), *E. coli* cells can be physically directed and concentrated near the electrode surface during the enrichment phase. While they yield a negligible impedance change, their physical presence creates nonspecific steric hindrance and local crowding in the mixed suspension that limit Gram-positive cells from binding to vancomycin receptors and thereby reduce the impedance shift. These results confirm the high specificity of the developed W_IDE biosensor toward Gram-positive bacteria while effectively suppressing nonspecific responses.

### 3.4. Reproducibility Study

The reproducibility of the pDEP-assisted wave-shaped biosensor was investigated by performing repeated measurements at a fixed concentration of *S. aureus* (10^5^ CFU/mL) using multiple electrodes within the W_IDE array. The impedance responses (Δ*Z*/*Z*_0_) exhibited minimal variation, as shown in Figure 7f, yielding a relative standard deviation (RSD) of 2.76%, which confirms excellent electrode-to-electrode reproducibility. Importantly, the application of pDEP did not compromise repeatability; instead, the controlled electric-field-driven transport of bacteria enabled uniform accumulation at the sensing surface, resulting in stable and consistent electrochemical signals.

### 3.5. Real-Sample (Skim Milk) Validation Using Passive and DEP-Assisted Approaches

**Figure 8:**
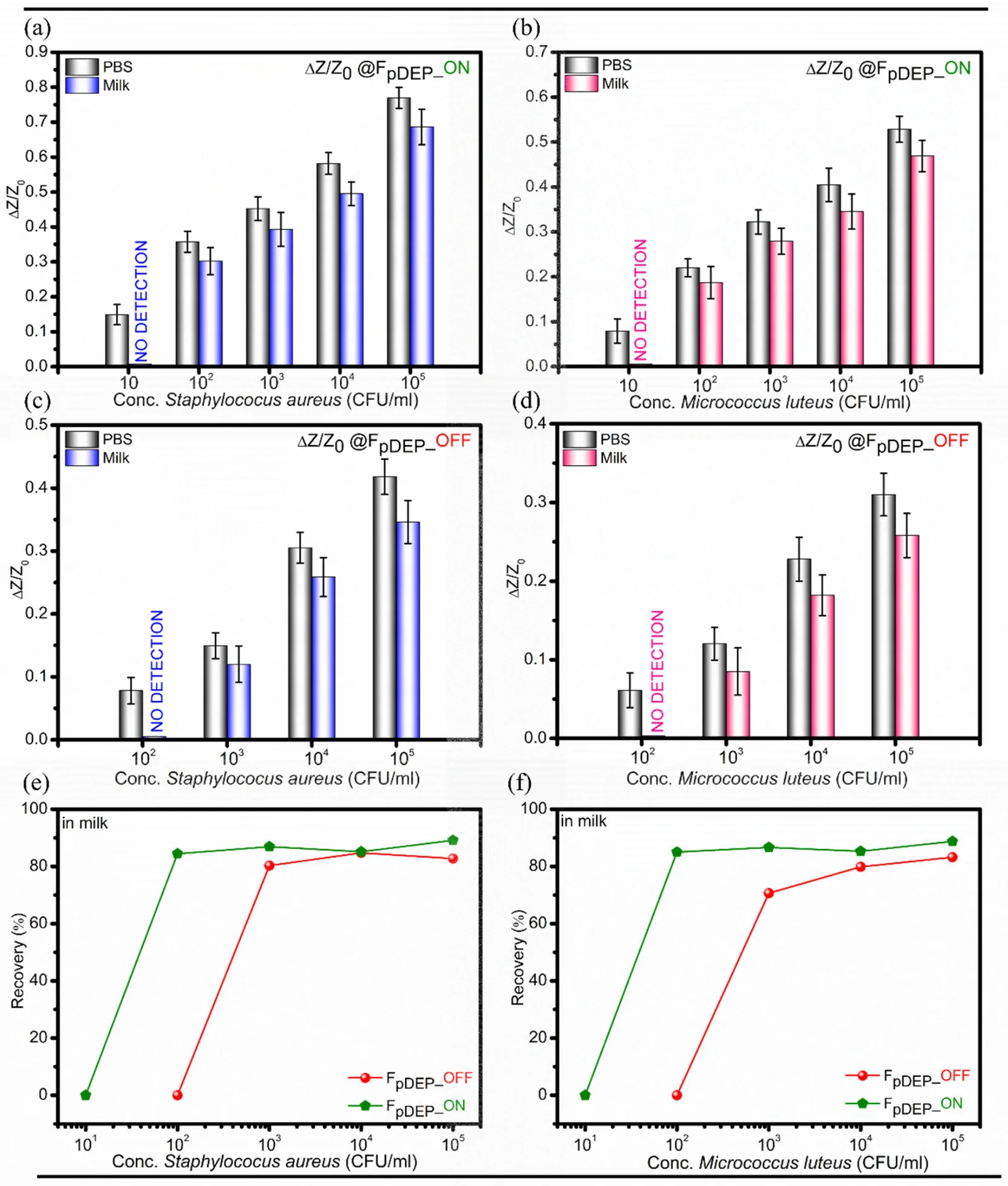
(**a**,**b**) Impedance changes (Δ*Z*/*Z*_0_) measured in skim milk and PBS for *S. aureus* and *M. luteus* under pDEP-assisted (F_pDEP__ON) operation. (**c**,**d**) Impedance changes (Δ*Z*/*Z*_0_) measured in skim milk and PBS for *S. aureus* and *M. luteus* under passive (F_pDEP__OFF) detection. (**e**,**f**) Recovery values of *S. aureus* and *M. luteus* in skim milk under pDEP-assisted (F_pDEP__ON) and passive (F_pDEP__OFF) detection. The data are presented as mean values (n = 3), with error bars indicating the standard deviation.

To evaluate the applicability of the developed biosensor in complex food matrices, skim milk samples spiked with known concentrations of *Staphylococcus aureus* and *Micrococcus luteus* were measured under both pDEP-assisted (F_pDEP__ON) and passive (F_pDEP__OFF) conditions. Skim milk was chosen as a representative food matrix due to its high conductivity, ionic strength, protein content, and fat residues, which are known to suppress bacterial polarizability and affect dielectrophoretic forces [58], thereby providing a practically relevant and complex medium for evaluating the performance of pDEP-assisted (F_pDEP__ON) as well as passive (F_pDEP__OFF) biosensing. Figure 8a–d compares the impedance change (Δ*Z*/*Z*_0_) obtained in skim milk and PBS for *S. aureus* and *M. luteus* under pDEP-assisted and passive conditions. For the pDEP-assisted condition (Figure 8a,b), Δ*Z*/*Z*_0_ in skim milk closely follows the same concentration-dependent trend observed in PBS despite the highly conductive and complex dairy matrix. However, the milk environment introduced a moderate signal reduction and increased the practical detection limit; *S. aureus* and *M. luteus* could only be detected from 10^2^ to 10^5^ CFU/mL, with 10 CFU/mL falling below the noise threshold. In contrast, under passive conditions (FpDEP_OFF) in skim milk, the impedance response (Δ*Z*/*Z*_0_) was further reduced, particularly at low bacterial concentrations, due to limited diffusion and matrix screening, as shown in Figure 8c,d. As a result, detectable signals were obtained only in the higher concentration range of 10^3^–10^5^ CFU/mL for both *S. aureus* and *M. luteus*. The quantitative impact of pDEP in milk was further confirmed by recovery analysis (Figure 8e,f), where bacterial recovery was calculated by comparing the response in spiked milk to that in PBS at identical concentrations. Accordingly, the recovery plots provide a quantitative representation of the impedance trends shown in Figure 8a–d. For pDEP-assisted detection, high and stable recoveries between ~85–90% were obtained for both *S. aureus* and *M. luteus* once the bacteria entered the detectable range. In contrast, under passive detection operation, no recovery was observed at low concentrations (10 and 10^2^ CFU/mL) and meaningful recoveries between ~70–84% were obtained only at higher concentrations (10^3^ and 10^5^ CFU/mL). These results highlight the important role of pDEP in maintaining sensitivity and extending the usable detection range of the biosensor in real food samples. It is worth noting that no significant protein fouling from the skim milk matrix was observed during testing. A gentle buffer wash was performed between runs without additional pretreatment or regeneration steps, and no detectable baseline drift was observed, indicating that protein fouling did not materially affect the sensor response under these short-term conditions. However, for practical translation to long-term continuous use, cumulative protein adsorption may become more relevant. This could be addressed through appropriate sample pretreatment, periodic electrode regeneration, or routine sensor replacement to mitigate cumulative fouling.

### 3.6. Comparison Study

The performance of the proposed pDEP-assisted biosensor platform was compared with representative studies reported in the literature, as summarized in Table 2. The developed platform demonstrates competitive or superior performance while offering several practical advantages. First, the FEM-optimized wave-shaped interdigitated electrode architecture provides enhanced electric field and bacterial capture, enabling sensitive detection of Gram-positive bacteria down to 10 CFU/mL within 30 min. Second, the biosensor allows detection of multiple Gram-positive bacterial species (*S. aureus and M. luteus*) using a single platform, highlighting its versatility. Moreover, the integration of positive dielectrophoresis with vancomycin-based biorecognition facilitates rapid enrichment and selective binding, supporting sensitive measurements even in complex matrices. The multichannel sensor array, coupled with a portable 16-channel electrochemical measurement system, enables sequential measurements with reduced analysis time, highlighting the potential of this platform for rapid bacterial monitoring.

## 4. Conclusions

This work establishes a combined numerical–experimental approach to design and evaluate a positive dielectrophoretic-assisted electrochemical biosensor for rapid and sensitive detection of Gram-positive bacteria. Finite-element modeling revealed that a wave-shaped interdigitated electrode (W_IDE) produces extended high-field regions and an enlarged effective capture area, resulting in a predicted bacterial capture efficiency of 19%, compared to 12% for conventional interdigitated electrodes (R_IDE) under positive DEP forces (F_pDEP__ON). The Experimental results confirmed that pDEP-assisted detection greatly accelerates bacterial accumulation at the sensor surface, allowing detection of *S. aureus* and *M. luteus* at concentrations as low as 10 CFU/mL within just 30 min, with a linear detection range of 10–10^5^ CFU/mL in PBS. In contrast, passive detection (F_pDEP__OFF) required 45 min and was limited to a narrower linear range of 10^2^–10^5^ CFU/mL. Evaluation in a complex food matrix (skim milk) further demonstrated the robustness of this approach: pDEP-assisted electrochemical detection preserved sensitivity, yielding a linear range of 10^2^–10^5^ CFU/mL for both *S. aureus* and *M. luteus*, with recoveries around 85–90%. Passive detection in skim milk, however, exhibited a narrower linear range of 10^3^–10^5^ CFU/mL with lower recoveries of 70–84% for both bacterial species. This integrated strategy provides a versatile platform for low-concentration bacterial sensing in real-world applications, including food safety monitoring, clinical diagnostics, and environmental surveillance. Future work will focus on extending the platform to a broader range of bacterial pathogens, including Gram-negative species, and complex real-world samples, while advancing multiplexed detection and integrating wireless multichannel electrochemical data acquisition to facilitate field deployment and commercial prototyping.

## Figures and Tables

**Table 1:** Physical properties and parameters used in the FEM simulations for dielectrophoretic modeling of bacterial particles.

Parameter	Value	Unit	Reference
$\varepsilon_{m\;}$	80	_	[47]
$\varepsilon_{p\;}$	70	_	[48]
$\sigma_{p}$	0.8	S/m	[48]
$\sigma_{m}$	0.16	S/m	[49]
$d_{p}$	1	µm	[50]
$r_{p}$	0.5	µm	[50]
$\rho_{p}$	1000	Kg/m^3^	[51]

**Table 2:** Comparative analysis of DEP-assisted biosensors for cell detection.

Target Cell	LOD	Enrichment Time	Principle	Sample Type	Ref.
*E. coli*	300 CFU/mL	1 min	pDEP-assisted bacterial capture and impedance detection	Drinking water	[59]
*Salmonella enterica*	1.0 CFU/mL	3 min	Magnetic bead–integrated DEP impedance detection	Chicken meat, milk	[60]
*S.aureus*, *E.coli*	10^3^ CFU/mL	2 min	DEP-based SWCNT functionalized electrochemical Immunosensor	0.1% peptone water	[61]
*Salmonella Typhimurium*	56 CFU/mL	30 min	pDEP-driven fluorescent nanoparticle immunodetection	DI water	[62]
*Salmonella enteritidis*	15 CFU/mL	5 min	Conventional lateral flow immunoassay for DEP trapping using SPEs	Field water	[63]
*Staphylococcus epidermidis*	3.5 × 10^5^ CFU/mL	20 min	Coupled long-range electrothermal flow to short-range DEP	Diluted PBS	[64]
*E. coli*	3.0 × 10^2^ CFU/mL	20 min	iSPR chips under positive-DEP conditions	DI water	[65]
*E. coli*	5.0 × 10^4^ CFU/mL	6 min	DEP enrichment coupled with impedance sensing	Synthetic chicken	[66]
*S. aureus*	36 CFU/mL	10 min	Magnetophoretic–DEP-assisted impedimetric detection	Homogenized cabbage	[67]
*S. aureus*	93 CFU/mL	30 min	pDEP-driven enrichment and aptamer-silica nanoparticles	DI water	[68]
*S. aureus*, *M. luteus*	10 CFU/mL, 10^2^ CFU/mL	30 min	Vancomycin immobilized interdigital wave-shaped biosensor under pDEP	PBS, skim milk	This work

## Data Availability

The original contributions presented in this study are included in the article. Further inquiries can be directed to the corresponding author.

## Supplementary Materials

The following supporting information can be downloaded at https://www.mdpi.com/article/10.3390/bios16090527/s1: Section S1: Reagents and chemicals; Section S2: Preparation of bacteria for SEM analysis; Figure S1: PCB images of the 16-channel portable electrochemical measurement system integrating the Em-Stat Pico potentiostat (a) Top view (b) Bottom view; Figure S2: Optical images of the experimental setup showing the wave-shaped interdigitated electrode (W_IDE) array mounted in a custom 3D-printed holder and electrically interfaced with our de-signed 16 channel electrochemical measurement system based on an EmStat Pico potentiostat; Table S1: List of components used in the electrochemical measurement system.
