# Supplementary material for: Electric-Field-Assisted Enrichment and Electrochemical Detection of Gram-Positive Bacteria Using an FEM-Guided Interdigitated Electrode Biosensor
Source: Biosensors (Basel). 2026 Sep 21;16(9):527. doi: 10.3390/bios16090527 (PMC13604522; doi:10.3390/bios16090527)
Supplement: Supplementary file 1 [file biosensors-16-00527-s001.zip › biosensors-4529573-supplementary.pdf]

biosensors

Supplementary

Electric-Field-Assisted Enrichment and Electrochemical Detection of Gram-Positive Bacteria Using an FEM-Guided Interdigitated Electrode Biosensor

Zeeshan [1,*], Naeem Iqbal [2,3] and Jaeyoung Choi [1,*]

[1] School of Computing, Gachon University, Seongnam-si 13120, Gyeonggi-do, Republic of Korea
[2] Centre for Secure Information Technologies, Queen's University Belfast, Belfast BT3 9DT, UK; n.iqbal@qub.ac.uk
[3] Momentum 1.0 Research, Queen's University Belfast, Belfast BT3 9DT, UK
* Correspondence: zeeshan@gachon.ac.kr (Z.); jychoi19@gachon.ac.kr (J.C.)

S1. Reagents and chemicals

The bacterial strains used in this work included *Staphylococcus aureus* (ATCC 12692), and *Micrococcus luteus* (ATCC 7468), sourced from the American Type Culture Collection (ATCC). Hydrogen hexachloroplatinate (IV) hexahydrate ($H_2PtCl_6{\cdot}6H_2O$) (≥ 37.50% Pt basis), Lead diacetate trihydrate (≥ 99%), Hydrochloric acid (HCl), Glutaraldehyde, Potassium ferrocyanide ($K_4[Fe(CN)_6]$), Potassium ferricyanide ($K_3[Fe(CN)_6]$), Potassium chloride (KCl), Vancomycin hydrochloride, 6-Mercapto hexanoic acid (90%), *N*-(3-(dimethylamino) propyl)-*N*-ethylcarbodiimide (EDC), N-hydroxysuccinimide (NHS, 98.0%) were purchased from Sigma-Aldrich (Seoul, Korea). Phosphate-buffered saline (PBS) and Bovine serum albumin (BSA) were obtained from Tech and Innovation (Shuncheon-si, Korea). Ethanol (extra pure, 94%) was purchased from OCI Company Ltd., (Seoul, Korea) and DI water was provided by the Water Purification System, PURESCIENCE (Seongnam-si, Korea).

S2. Preparation of bacteria for SEM analysis

Bacterial cultures were collected by centrifugation at 10,000 rpm for 5 min to obtain cell pellets. The pellets were washed twice with phosphate-buffered saline (PBS) by centrifugation at 10,000 rpm for 2 min. The washed bacterial cells were then fixed by incubation in 0.5% glutaraldehyde prepared in PBS (pH 7.4) for 2 h at room temperature. After fixation, the samples were rinsed twice with PBS using centrifugation at 10,000 rpm for 2 min to eliminate excess fixative. The fixed bacterial suspension was subsequently incubated on the fabricated electrode surface. Prior to SEM imaging, the electrode was coated by Pt sputtering for 120 s, and morphological characterization was carried out using scanning electron microscopy.

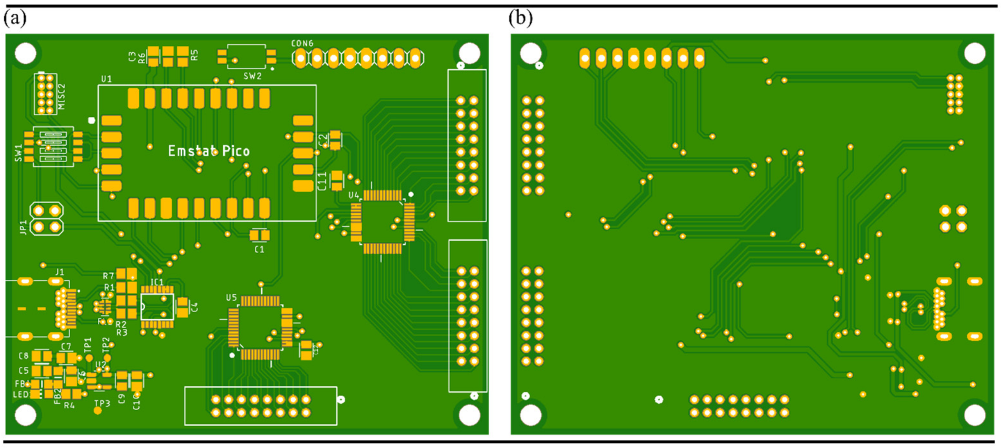

Figure S1. PCB images of the 16-channel portable electrochemical measurement system integrating the EmStat Pico potentiostat (a) Top view (b)Bottom view.

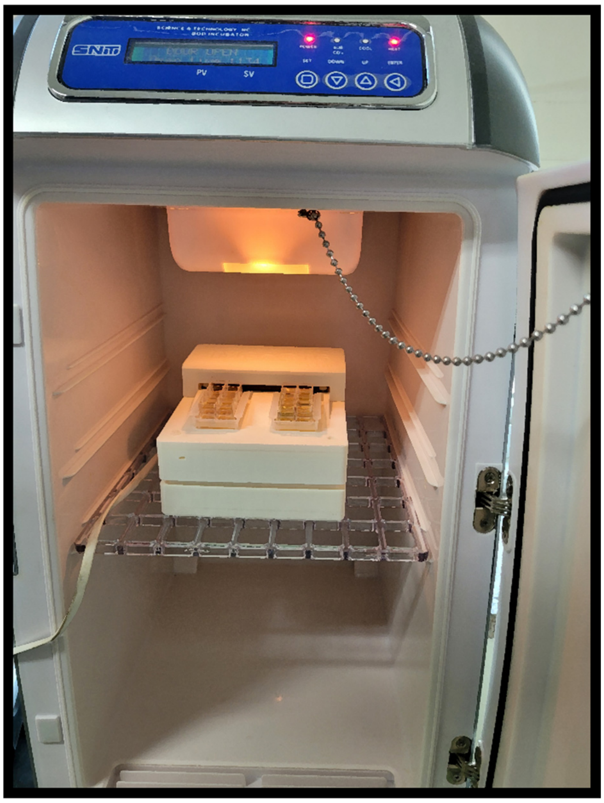

Figure S2. Optical images of the experimental setup showing the wave-shaped interdigitated electrode (W_IDE) array mounted in a custom 3D-printed holder and electrically interfaced with our designed 16 channel electrochemical measurement system based on an EmStat Pico potentiostat.

Table S1. List of components used in the electrochemical measurement system.

Qty	Value	Device	Parts	Description
4	100nF 10V X5R	C_CHIP-0805(2012-METRIC)	C4, C7, C11, C12	Capacitor
1	10nF 10V X5R	C_CHIP-0805(2012-METRIC)	C9	Capacitor
3	10uF 10V X5R	C_CHIP-0805(2012-METRIC)	C3, C6, C10	Capacitor
1	1571983-5	1571983-5	SW1	SWITCH SLIDE
3	1k	R-US_R0805	R4, R5, R6	RESISTOR
2	1uF 10V X5R	C_CHIP-0805(2012-METRIC)	C5, C8	Capacitor
1	218-2LPSTRF	218-2LPSTRF	SW2	Switch DIP OFF
1	632723100011	632723100011	J1	USB-C
2	ADG726BSUZ	ADG726BSUZ	U4, U5	Multiplexers
3	AWHW2-16G-0202	AWHW2-16G-0202	J2, J3, J4	Headers, Male Pins
1	EMSTATPICOV1.3	EMSTATPICOV1.3	U1	EmStat Pico
1	FT230XS-US	FT230XS-US	IC1	USB to UART IC
1	PINHD-2X2	PINHD-2X2	JP1	PIN HEADER
1	LEDCHIP-LED0805	LEDCHIP-LED0805	LED1	LED
1	LT1761ES5-3.3#PBF	LT1761ES5-3.3#PBF	U2	Voltage Regulator IC
1	SWD	2X5-PTH-1.27MM	MISC2	Cortex Debug Connector
1	ECMF02-4CMX8	ECMF02-4CMX8	FL1	ESD Protection
2	STANDARD	RC-MASTER-SMD_FB_0805MP	FB1, FB2	THERMALLOSS FERRITE BEAD
1	PINHD-1X8	PINHD-1X8	CON6	PIN HEADER
2	C_CHIP-0805	C_CHIP-0805	C1, C2	Capacitor
2	27R	R-US_R0805	R2, R3	RESISTOR
3	TPTP10R	TPTP10R	TP1, TP2, TP3	Test pad
